# Prevalence and Associated Factors of Frailty and Mortality in Patients with End-Stage Renal Disease Undergoing Hemodialysis: A Systematic Review and Meta-Analysis

**DOI:** 10.3390/ijerph18073471

**Published:** 2021-03-27

**Authors:** Hyeon-Ju Lee, Youn-Jung Son

**Affiliations:** 1 Department of Nursing, Tongmyong University, Busan 48520, Korea; lhj209@tu.ac.kr; 2 Red Cross College of Nursing, Chung-Ang University, Seoul 06974, Korea

**Keywords:** frailty, kidney disease, hemodialysis, mortality, risk factor, systematic review

## Abstract

Hemodialysis is the most common type of treatment for end-stage renal disease (ESRD). Frailty is associated with poor outcomes such as higher mortality. ESRD patients have a higher prevalence of frailty. This systematic review and meta-analysis aimed to identify the prevalence and associated factors of frailty and examine whether it is a predictor of mortality among ESRD patients undergoing hemodialysis. Five electronic databases including PubMed, Embase, CINAHL, Web of Science, and Cochrane Library were searched for relevant studies up to 30 November 2020. A total of 752 articles were found, and seven studies with 2604 participants in total were included in the final analysis. The pooled prevalence of frailty in patients with ESRD undergoing hemodialysis was 46% (95% Confidence interval (CI) 34.2−58.3%). Advanced age, female sex, and the presence of diabetes mellitus increased the risk of frailty in ESRD patients undergoing hemodialysis. Our main finding showed that patients with frailty had a greater risk of all-cause mortality compared with those without (hazard ratio (HR): 2.02, 95% CI: 1.65−2.48). To improve ESRD patient outcomes, healthcare professionals need to assess the frailty of older ESRD patients, particularly by considering gender and comorbidities. Comprehensive frailty screening tools for ESRD patients on hemodialysis need to be developed.

## 1. Introduction

Chronic kidney disease (CKD) is a progressive loss in kidney function and an irreversible clinical syndrome that is characterized by the kidney’s failure to filter waste products and remove excessive fluid from the body [1]. The global prevalence of CKD has been reported to be 8–16% [2]. End-stage renal disease (ESRD) represents a serious public health problem fueled by aging populations and a pandemic of chronic noncommunicable diseases [3,4]. Although overall mortality rate of the ESRD population is improving, the mortality rate has been reported to be up to 30% within the first year of transition from CKD to ESRD [5,6]. In particular, rapid progression of CKD is associated with worse clinical outcomes, such as cardiovascular events and death [6].

To maintain daily life activities, renal replacement therapy is commonly used for ESRD in clinical practice, including hemodialysis, peritoneal dialysis, and kidney transplantation [7]. Although kidney transplantation is the optimal choice of ESRD treatment, challenges such as immunosuppression and organ donor shortage remain an issue [8,9]. Therefore, ESRD necessitates management using dialysis to postpone imminent death [9,10]. Among advanced CKD or ESRD treatment modalities, hemodialysis was found to be the primary approach of dialysis treatment for kidney failure, and around 90% of all patients undergoing dialysis received hemodialysis [11,12]. According to a recent, nationwide cohort study [13], hemodialysis patients had a lower adjusted risk of death compared with those on peritoneal dialysis. However, the hemodialysis patients still have higher mortality as well as multimorbidity and hospitalizations than the general population [14]. According to previous studies, several factors such as age, presence of diabetes mellitus, history of cardiovascular events, and elevated high-sensitivity C-reactive protein were reported as risk factors for mortality in hemodialysis patients [14,15,16]. However, the risk factors for mortality in ESRD patients undergoing hemodialysis are not fully understood.

Frailty is characterized by reduced cognitive and physiologic reserves which lead to greater vulnerability [17]. Frailty is prevalent in patients with all stages of CKD and two-thirds of patients with ESRD [18,19]. ESRD patients with frailty are vulnerable to adverse outcomes including an increased risk of disability, hospitalization, and mortality [19]. Two previous studies have also demonstrated the relationship between frailty in hemodialysis and adverse outcome, such as death [20,21]. Moreover, despite improved care and innovative technological advancements in hemodialysis, the mortality of patients with ESRD is 10−30 times greater than that of the general population [22]. Although frailty before ESRD has been evaluated as a risk factor for short- and long-term mortality in adults with ESRD, the impact of preexisting or coexisting frailty on mortality after ESRD among adult patients of all ages is unclear. Hence, it is meaningful to synthesize and analyze whether frailty has an impact on mortality among adults with ESRD undergoing hemodialysis. Strategies to reduce the risk of mortality associated with hemodialysis among ESRD patients should continue to be a high clinical priority. In addition, identifying prevalence and risk factors of frailty in ESRD patients undergoing hemodialysis may be meaningful for creating strategies designed to reduce the burden on patients and their family members.

A previous systematic review [23] investigated the assessment for frailty in pre-dialysis patients and reported that it is associated with CKD. This review highlighted that it is difficult to examine the causal relationship between the factors identified and frailty, as well as the association between frailty and mortality [23]. To our knowledge, careful analysis of the impact of frailty on mortality of ESRD patients undergoing hemodialysis has not yet been performed. Therefore, this study aimed to investigate whether frailty is associated with mortality in hemodialysis patients with ESRD based on a review of cohort studies. In addition, we aimed to explore the prevalence and associated factors of frailty in ESRD patients on hemodialysis. 

## 2. Materials and Methods

### 2.1. Search Strategies

We conducted a systematic review, prospectively registered on PROSPERO (ID: CRD42021228373). This systematic review was performed based on the Preferred Reporting Items for Systematic Reviews and Meta-Analysis (PRISMA) guidelines [24]. We organized the research question according to the “PICO” format [25], which was: What is the prevalence and associated factors of frailty (Interest) and the association of frailty and mortality (Outcome) in patients with ESRD undergoing hemodialysis (Patient), compared with patients without hemodialysis-dependent ESRD (Comparator)? Our systematic review was conducted by reviewing the literature in electronic databases such as PubMed, Embase, CINAHL, Web of Science, and Cochrane Library for cohort studies published until 30 November 2020. 

Medical Subject Heading (MeSH) and non-MeSH keywords were used in the search strategy based on our research question: “frailty or frail”, “dialysis or hemodialysis”, “chronic kidney disease or CKD or end-stage renal disease or ESRD”, and “mortality or death or outcome”. Additional searches were conducted on Google Scholar and in grey literature sources such as conferences and government websites. The reference lists of full-text articles were also examined. 

### 2.2. Study Selection

From the retrieved results, two researchers (Y.J.S. and H.J.L.) screened all abstracts to verify potentially relevant articles for this review. Then, the full text of studies were independently assessed to verify articles that were qualified to be included. Any disagreement encountered was settled by discussion. In this review, the inclusion criteria were as follows: (1) studies reporting the association of frailty and mortality including the prevalence and associated factors of frailty among patients undergoing hemodialysis (≥18 years of age), (2) cohort studies (prospective or retrospective), (3) reporting of the hazard ratio (HR) or odds ratio (OR) and standardized mean difference (SMD) with 95% confidence interval (CI) or eligible data to calculate these figures, and (4) full text published in English in peer-reviewed journals. The exclusion criteria were: (1) case-control studies or cross-sectional studies, clinical trials, reviews, editorials, letters, and abstracts, and (2) patients undergoing peritoneal dialysis or kidney transplantation.

### 2.3. Data Extraction and Synthesis

Data were extracted by two researchers (Y.J.S. and H.J.L.) using a prespecified form. These data included the first author’s last name, publication year, country, study design, follow-up period, frailty scale, definition of frailty, participants’ characteristics (e.g., sample size for frail and non-frail patients, mean age, and sex), prevalence of frailty, outcome, and adjustments for covariates. 

### 2.4. Data Analysis

Among the risk factors associated with frailty in patients with ESRD undergoing hemodialysis, the binomial variables are presented as OR with 95% CI, and the continuous variable is presented as SMD with 95% CI. The all-cause mortality in patients with ESRD undergoing hemodialysis is presented as HR with 95% CI. Statistical heterogeneity in the results was calculated by Q statistics (*p* < 0.05 considered statistically significant) and quantified using I^2^ statistics. A value of I^2^ indicating 25%, 50%, or >75% heterogeneity was considered as low, moderate, or high heterogeneity, respectively [26]. If moderate or high heterogeneity was confirmed, we utilized a random effects model to pool outcomes. Conversely, if low heterogeneity was confirmed, we utilized a fixed effects model [27]. Publication bias was assessed using Egger’s linear regression tests [28]. Meta-analysis was conducted using Comprehensive Meta-Analysis software (version 3.0; Biostat, Englewood, NJ, USA).

### 2.5. Quality Assessment of the Studies Included

The Newcastle–Ottawa Scale (NOS) was used for assessing the quality of cohort studies [29]. This scale has four items that examine selection categories, one that examines comparability, and three items that examine outcome categories. Each item is awarded a maximum of one star within selection and outcome categories. A maximum of two stars can be given for comparability. Studies scoring ≥7 were judged to be of good quality, 5−6 as fair quality, and <5 as poor quality. Two researchers (Y.J.S. and H.J.L.) independently judged the studies for risk of bias. Any discrepancies encountered were settled by discussion.

## 3. Results

### 3.1. Study Selection

**Figure 1:**
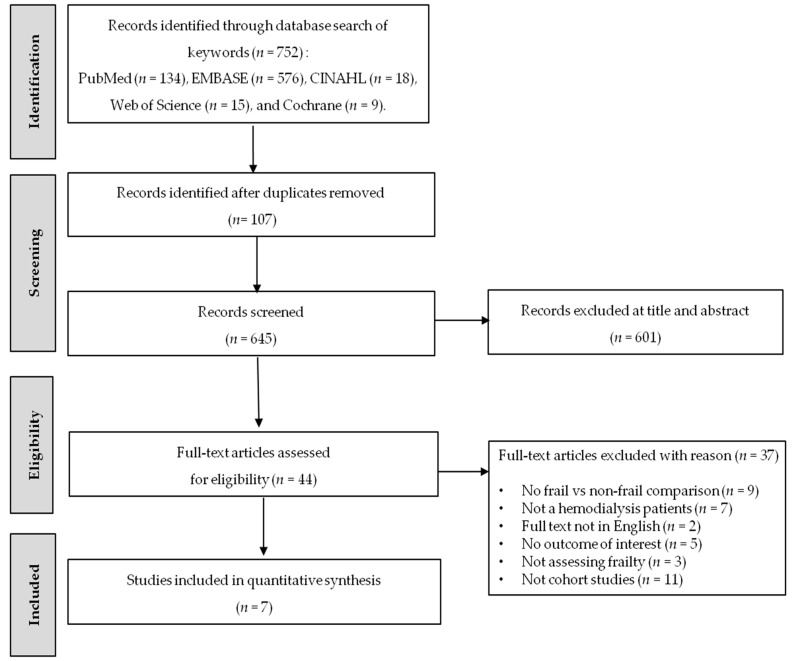
Preferred Reporting Items for Systematic Reviews and Meta-Analysis (PRISMA) flow diagram for study selection.

The PRISMA selection process is shown in Figure 1. A total of 752 studies were retrieved through database searches. After removal of duplicates, we reviewed the title and abstract of 645 relevant studies. Of these articles, 601 were excluded, leaving 44 articles for full-text review. A total 37 articles were excluded for the reasons described in Figure 1, which finally left seven articles for the meta-analysis.

### 3.2. Characteristics of the Included Studies

The characteristics of the included studies are described in Table 1. According to study design, the types of cohort studies in this review included six prospective cohort studies [20,30,31,32,33,34] and one retrospective cohort study [35]. The follow-up periods ranged from 12 to 36 months. In terms of assessment tools for measuring frailty, six studies used the Fried phenotype, determining frailty if three or more of the five components were present [30,31,32,33,34,35]. One study using the Edmonton Frail Scale identified frailty when a score of more than 8 out of 17 was obtained [20]. The cohort size of the studies ranged from 117 to 762, with a total of 2604 patients, and the number of frailty cases ranged from 63 to 240, with a total of 1036. The mean ages of the study populations ranged from 44.9 to 78.1 years. All the studies achieved a NOS methodological quality score ranged 7 to 8, which means good quality. 

### 3.3. Prevalence of Frailty in Patients Undergoing Hemodialysis

**Figure 2:**
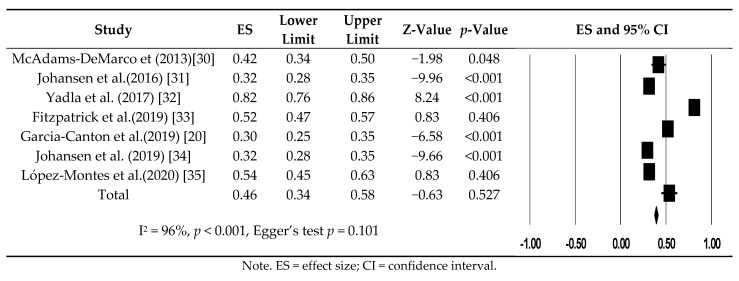
Forest plot of prevalence frailty in patients undergoing hemodialysis.

The prevalence of frailty ranged from 29.6% to 81.5% among all studies. The overall prevalence of frailty in patients with ESRD undergoing hemodialysis was 46.0% (95% CI: 34.2−58.3%). A random effects model was utilized for analysis because there was heterogeneity between them (I^2^ = 96%, *p* < 0.001) (Figure 2).

### 3.4. Risk Factors for Frailty in Hemodialysis Patients

We identified 11 risk factors for frailty among patients with ESRD undergoing hemodialysis (Table 2). However, only three factors including advanced age, female sex, and diabetes mellitus were significantly associated with frailty.

#### 3.4.1. Age

Six studies [29,30,31,32,33,34] assessed the association between age and frailty in ESRD patients undergoing hemodialysis. We used a random effects model for these studies because there was heterogeneity between them (I^2^ = 72%, *p* = 0.003). The results showed that advanced age was a risk factor for frailty in patients undergoing hemodialysis (SMD: 0.43 years; 95% CI: 0.24−0.61) (Table 2). 

#### 3.4.2. Female Sex

All studies included in this review identified an association between sex of the patient and frailty in ESRD patients undergoing hemodialysis. A random effects model was utilized to analyze the studies because they were heterogenous (I^2^ = 71%, *p* = 0.002). The results indicated that being female was a risk factor for frailty in ESRD patients undergoing hemodialysis (OR: 1.89; 95% CI: 1.33−2.67) (Table 2).

#### 3.4.3. Diabetes Mellitus 

All studies included in this review investigated the association between diabetes mellitus and frailty in ESRD patients undergoing hemodialysis. A random effects model was utilized to analyze these seven studies because they were heterogenous (I^2^ = 73%, *p* = 0.001). The results showed that diabetes mellitus was a risk factor for frailty in ESRD patients undergoing hemodialysis (OR: 2.42; 95% CI: 1.68−3.49) (Table 2).

### 3.5. All-Cause Mortality for Hemodialysis Patients with Frailty

**Figure 3:**
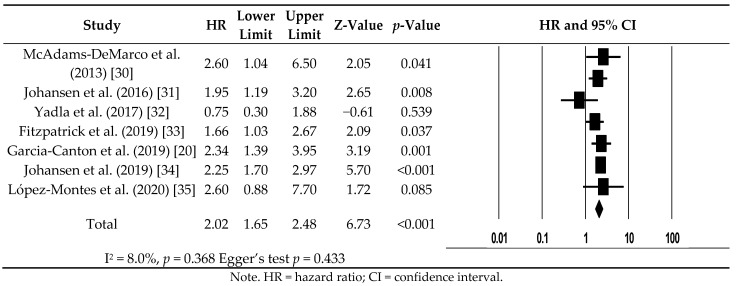
Forest plot of all-cause mortality for hemodialysis patients with frailty.

All studies included in this review utilized a fixed effects model because there was low heterogeneity (I^2^ = 8%, *p* = 0.368). Compared to hemodialysis patients with non-frailty, hemodialysis patients with frailty had a greater all-cause mortality risk (HR: 2.02, 95% CI: 1.65−2.48) (Figure 3).

## 4. Discussion

Our findings demonstrate that frailty increases the risk of all-cause mortality in ESRD patients undergoing hemodialysis, which is similar to previous reviews [18,36]. Frailty is a multidimensional syndrome that leads to decline in activity and inadequate response to health stressors due to sarcopenia, vulnerability, and impaired endurance [18]. Particularly, patients undergoing hemodialysis inevitably experience amino acid and protein losses through hemodialysis [37,38]. In addition, ESRD itself is likely to lead to frailty, due to insulin resistance, chronic inflammation, and vascular calcification resulting in loss of musculoskeletal mass [39,40,41]. For these reasons, ESRD patients on hemodialysis have an increased risk of frailty due to reduced protein reserves and body energy and decreased strength, which can lead to difficulties in self-care ability as a key component of long-term survival in renal care [7,19]. However, many of the mechanisms by which frailty leads to an increase in mortality are still not well defined. Further research is required to determine the mechanisms by which frailty increases the risk of mortality. 

Importantly, this review revealed that the prevalence of frailty in ESRD patients on hemodialysis was approximately 43%. Our finding was similar to a recent meta-analysis [42] that showed a 34% prevalence of frailty in patients undergoing hemodialysis. A systematic review by Chowdhury et al. also confirmed that the prevalence of frailty in dialysis patients was higher compared to non-dialysis patients [43]. Therefore, early assessment of frailty in patients undergoing hemodialysis is a vital factor for preventing the risk of mortality as well as disability and unplanned rehospitalization. Moreover, we found that there was a difference in the prevalence according to the type of assessment tool used to measure frailty. In our review, the most common screening tool for frailty was the Fried phenotype, with prevalence of frailty ranging from 31.0−81.5% [30,31,32,33,34,35]. Only one study used the Edmonton Frail Scale to measure frailty and found that the prevalence of frailty was 26%, lower than that found using the Fried phenotype [20]. The Fried phenotype is the most widely used validated method for frailty assessment [43]. However, this tool focuses on physiological frailty and has limitations for assessing the severity of frailty in a population with a high prevalence of frailty [44,45]. Whereas the Edmonton Frail Scale is a multiple domain tool that assesses various dimensions of frailty, including cognitive state, degree of dependence, social support, a physiological dimension, and a psychological dimension [46]. However, the Edmonton Frail Scale has only been used in a few studies [47,48]. Assessments of frailty for hemodialysis patients should include the risk factors related to its incidence and progression and should also be assessed with an expanded concept that includes not only physical but also psychological and cognitive frailty [17,19]. Therefore, it is necessary to develop a standardized assessment tool for screening frailty in patients undergoing hemodialysis.

Our findings show that advanced age, female sex, and presence of diabetes mellitus were risk factors for the progression and development of frailty. Moreover, this review found that older patients undergoing hemodialysis were more at risk of frailty. This result was consistent with recent studies showing a close correlation between frailty and aging [49,50]. Aging causes conditions such as chronic diseases, depressive symptoms, and declined cognitive and functional capacity, and aging increases the likelihood of developing frailty [51]. Particularly, ESRD may accelerate the aging process in patients due to uremic toxins, inflammations, protein energy waste, and oxidative stress [52]. Therefore, health professionals should pay close attention to older ESRD patients undergoing hemodialysis and take actions to prevent the development and progression of frailty. Meanwhile, in the present study, the highest prevalence of frailty was confirmed in the population with a mean age of 44 years [32], indicating the need for further investigation to determine causality between younger age and frailty in ESRD patients undergoing hemodialysis. Females also had higher rates of frailty in ESRD patients on hemodialysis. This result is in line with a previous meta-analysis examining frailty by sex; female patients were found to have a higher degree of frailty than male patients, across all age groups [53]. In female older adults with frailty, there are multiple anabolic hormonal deficiencies, of which the declining levels of testosterone is correlated with frailty [54,55]. Hence, health professionals are required to explore gender or sex differences in hemodialysis patients’ frailty status, which can be helpful for developing an individualized or patient-centered approach for improving health outcomes, including quality of life. Diabetes mellitus, which can increase the risk of frailty, has long been known as the leading cause of ESRD [56]. Our findings support the results from previous studies showing that diabetes mellitus was associated with frailty in patients undergoing hemodialysis. Kakio et al. demonstrated that diabetic kidney disease patients are significantly more at risk of frailty than non-diabetic CKD patients [57]. Diabetic patients are more at risk because of the combination of neuropathy with physiological dysfunction, impaired cognitive function resulting from cerebrovascular disease or brain degeneration, inflammation mechanisms, and the loss of self-care capacity [58,59]. Therefore, hemodialysis patients with diabetes mellitus should be classified as a risk group for developing frailty, requiring more careful and specific assessment and different treatment strategies to relieve or prevent their physiological and cognitive dysfunction. 

Although ESRD is a major global health problem, six of the seven studies included in this review were performed in Western countries for example, the United States of America and Spain. According to a study spanning 142 countries, the population of ESRD patients is increasing worldwide, with the highest rate observed in the Western Pacific Region [60]. Especially, the number of ESRD patients who need dialysis treatment in Asia is growing at a higher rate than other parts of the world [61]. Therefore, future studies for the prevention of frailty and mortality in ESRD patients undergoing hemodialysis should be conducted using large-scale international comparisons. We pooled adjusted risk estimates in this meta-analysis. In each individual study, the age and sex were adjusted in common, but other risk factors that could affect all-cause mortality, such as comorbidities, were not adjusted in common in all studies. Future research should adjust the risk factors that may affect mortality in ESRD patients in order to more clearly establish the association of frailty with all-cause mortality. 

Our study has several limitations. First, the meta-analysis of the prevalence of frailty showed high heterogeneity, but the cause of heterogeneity was not found due to the small number of studies in the final analysis. Second, we could not determine the psychological or behavioral risk factors in this review. Therefore, further studies are needed to consider the impact of psychological or behavioral factors such as cognitive function, depression, and nutritional patterns on frailty and its clinical outcomes in patients undergoing hemodialysis. Third, future studies should develop a validated screening tool for assessing the frailty of ESRD patients, which could help to predict accurate rates of frailty prevalence for this population. Finally, this review did not include studies published in languages other than English. 

## 5. Conclusions

This review emphasized that frailty is a significant predictor of all-cause mortality in patients with ESRD undergoing hemodialysis. Healthcare professionals should develop a standardized tool for assessing renal-specific frailty and design individualized support programs to improve health outcomes for patients considering factors such as advanced age, sex, and comorbidities. Our results need to be confirmed through large multicenter cohort studies on the impact of frailty on poor health outcomes. 

## Figures and Tables

**Table 1:** Characteristics of studies included (*n* = 7).

Author (Year)/ Country	Study Design	Follow- Up Period (Months)	Frailty Scale	Definition of Frailty	Sample Characteristics		Prevalence of Frailty (%)	Outcome	Adjustments for Covariates	Quality Score
					Frail	Non-Frail				
McAdams-DeMarco et al. (2013)/USA [30]	Prospective	36	Fried phenotype	Components ≥3	*n* = 61 Mean age: 62.9 ± 12.9 (years) Male: 28 (45.9%)	*n* = 85 Mean age: 58.6 ± 13.5 (years) Male: 50 (58.8%)	41.8	All-cause mortality	Age, sex, comorbidity, disability	8
Johansen et al. (2016)/USA [31]	Prospective	20	Fried phenotype	Components ≥3	*n* = 240 Mean age: 63.5 ± 13.1 (years) Male: 130 (54.2%)	*n* = 522 Mean age: 54.1 ± 14.4 (years) Male: 322 (61.7%)	31.0	All-cause mortality	Age, sex, race, BMI, diabetes, heart failure, coronary artery disease, serum albumin, CRP, dialysis via a catheter	8
Yadla et al. (2017)/India [32]	Prospective	12	Fried phenotype	Components ≥3	*n* = 167 Mean age: 50.0 ± 13.3 (years) Male: 112 (67.1%)	*n* = 38 Mean age: 50.0 ± 13.3 (years) Male: 30 (78.9%)	81.5	All-cause mortality	Not adjusted	8
Fitzpatrick et al. (2019)/USA [33]	Prospective	Unreported	Fried phenotype	Components ≥3	*n* = 193 Mean age: 57.2 ± 13.5 (years) Male: 112 (58.0%)	*n* = 177 Mean age: 52.3 ± 12.1 (years) Male: 104 (58.8%)	52.0	All-cause mortality	Age, sex, race, CCI, serum albumin	7
Garcia-Canton et al. (2019)/Spain [20]	Prospective	29	Edmonton Frail Scale	Scoring ≥8	*n* = 82 Median age: 71.0 (years) Male: 42 (52.4%)	*n* = 195 Median age: 63.5 (years) Male: 139 (71.3%)	29.6	All-cause mortality	CCI	8
Johansen et al. (2019)/USA [34]	Prospective	24	Fried phenotype	Components ≥3	*n* = 230 Mean age: 62.9 ± 12.2 (years) Male: 93 (40.4%)	*n* = 497 Mean age: 54.6 ± 13.6 (years) Male: 306 (61.6%)	31.6	All-cause mortality	Age, sex, race, ethnicity, BMI, diabetes, atherosclerotic heart disease, heart failure, dialysis via a catheter, serum albumin concentration	8
López-Montes et al. (2020)/Spain [35]	Retrospective	12	Fried phenotype	Components ≥3	*n* = 63 Mean age: 78.6 ± 3.8 (years) Male: 29 (46.0%)	*n* = 54 Mean age: 77.4 ± 4.3 (years) Male: 45 (83.3%)	53.8	All-cause mortality	Age, sex, BMI, CCI	8

Note. BMI = body mass index; CRP = C-reactive protein; CCI = Charlson comorbidity index.

**Table 2:** Risk factors for frailty in hemodialysis patients.

Risk Factors	No. of Studies	No. of Participants	OR/SMD	95% CI	I^2^ (%)	*p*-Value	Egger’s Test p
Demographic characteristics							
Age (years)	6	1787	0.43 *	0.24−0.61	72	0.003	0.018
Sex (female)	7	2604	1.89	1.33 −2.67	71	0.002	0.395
Smoking, yes	3	721	1.39	0.58−3.32	80	0.005	0.186
Comorbidities							
Diabetes mellitus, yes	7	2604	2.42	1.68−3.49	73	0.001	0.108
Hypertension, yes	3	721	2.16	0.46−10.04	82	0.003	0.472
CAD, yes	3	1249	0.96	0.63−1.46	57	0.098	0.668
PVD, yes	5	1600	1.87	0.81−4.29	68	<0.001	0.240
HF, yes	4	1483	1.35	0.92−2.00	57	0.070	0.588
CVA or TIA, yes	4	1454	1.96	0.93−4.17	73	0.011	0.161
COPD, yes	3	633	1.43	0.98−2.09	0	0.835	0.532
Cancer, yes	2	516	1.35	0.48−3.84	68	0.077	NA

Note. * SMD = standardized mean difference; OR = odds ratio; CI = confidence interval; CAD = coronary artery disease; PVD = peripheral vascular disease; HF = heart failure; CVA = cerebral vascular disease; TIA = transient ischemic attack; COPD = chronic obstructive pulmonary disease.

## Data Availability

The datasets used and/or analyzed during the current study are available from the corresponding author on reasonable request.
